# The second French plan for rare diseases 2011-2014

**DOI:** 10.1186/1750-1172-7-S2-A4

**Published:** 2012-11-22

**Authors:** Alain Garcia, Christel Nourissier

**Affiliations:** 1 Rare Diseases National Plan, IGAS, 39-43 quai André Citroën, 75739 Paris Cedex 15, France; 2 EURORDIS Rare Diseases Europe, Plateforme Maladies rares, 102 rue Didot, 75014 Paris, France

## 

Rare diseases are very complex and require comprehensive strategic planning. Because health and social services were not well adapted to the needs of those most vulnerable patients, a first National Plan for Rare Diseases has been implemented in France between 2009 and 2010, acknowledging the specificities of rare diseases. Information was developed for patients, professionals and the general public (in Orphanet database and Maladies Rares Info Service helpline), access to high quality care and treatment was facilitated with the designation of 131 centres of reference at National level, and 502 centres of competence at regional level, and the coordination and funding of research was improved, with a 200 million Euros budget overall.

After a thorough evaluation, the second French Plan consolidates previous achievements, and reinforces European and international cooperation. The aim is to diagnose and cover each and every disease and patient. 20 university laboratories of genetics have been equipped with Next Generation Sequencing technology for clinical use. Research, public health and social authorities, health agencies, patients associations, experts, scientific societies are working together to improve the evaluation of the centers of expertise and regroup them into 20 to 30 networks, to be further integrated in European reference networks. Centers coordinate diagnosis, provision of health and social care, training, they write National Protocols for Diagnosis and Care, collect data, conduct clinical trials. A Foundation for research on rare diseases including a National data bank has been created.

Thanks to the work of patient organizations and health professionals raising awareness about rare diseases, to a close and exemplary collaboration between them and public authorities, the 3 axes, 15 measures, 47 actions, and more than 200 Millions € budget of the Second Plan set up rare diseases as a model for structuring health care provision and research, as well as a model for a tolerant society and an open democracy: 8000 rare diseases, an exemplary model.

